# 引言

**DOI:** 10.3724/SP.J.1123.2022.06019

**Published:** 2022-08-08

**Authors:** Feng ZHANG

**Affiliations:** 中国检验检疫科学研究院

食品安全事关人民群众身体健康和生命安全,事关经济健康发展与社会和谐稳定。优异的分离分析技术是保障食品安全的重要手段,食品基质复杂,目标物种类繁多,且含量水平低,加之近年来食品安全有害物隐蔽性越来越强,通过化学性修饰等手段使目标物的形态多样等,给食品安全分离分析带来极大的挑战。

近些年,我国食品安全分析技术发展较快,在提高方法灵敏度、准确度、检测通量等方面取得了非常大的进步,尤其在纳米材料、多孔有机骨架材料、金属有机框架材料、智能材料等基于新型功能性吸附材料的样品前处理技术,智能化、在线分析、实时分析、无损检测、快速检测、可视化检测、绿色检测等分析技术以及基于组学的食品真伪识别和风险技术等方面的研究创新不断涌现,极大地推动了食品安全分离分析技术的发展和食品安全检测技术能力的提升。

有鉴于此,我们组织了“食品安全分离分析”系列专辑,分为功能性吸附材料、食品安全高分辨分离分析技术、食品“组学”鉴别技术等主要内容,诚挚邀请了国内食品安全分离分析研究与应用相关的高等院校、科研院所研究人员为本刊撰稿。经过专家严格评审,筛选优秀的稿件以专刊或专栏的形式刊出,以期探讨并展示该领域的前沿技术及其进展。希望本刊能为读者带来启迪,碰撞出智慧的火花,不断完善食品安全分析技术,提升我国食品安全检验检测技术水平。

中国检验检疫科学研究院

张 峰 研究员


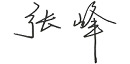


2022年6月28日

